# Self-Amplifying mRNA Vaccines Expressing Multiple Conserved Influenza Antigens Confer Protection against Homologous and Heterosubtypic Viral Challenge

**DOI:** 10.1371/journal.pone.0161193

**Published:** 2016-08-15

**Authors:** Diletta Magini, Cinzia Giovani, Simona Mangiavacchi, Silvia Maccari, Raffaella Cecchi, Jeffrey B. Ulmer, Ennio De Gregorio, Andrew J. Geall, Michela Brazzoli, Sylvie Bertholet

**Affiliations:** 1 Novartis Vaccines, S.r.l., Siena, Italy; 2 Dipartimento di Biotecnologie, Chimica e Farmacia, Università degli Studi di Siena, Siena, Italy; 3 Novartis Vaccines Inc., Cambridge, MA, United States of America; Deakin University, AUSTRALIA

## Abstract

Current hemagglutinin (HA)-based seasonal influenza vaccines induce vaccine strain-specific neutralizing antibodies that usually fail to provide protection against mismatched circulating viruses. Inclusion in the vaccine of highly conserved internal proteins such as the nucleoprotein (NP) and the matrix protein 1 (M1) was shown previously to increase vaccine efficacy by eliciting cross-reactive T-cells. However, appropriate delivery systems are required for efficient priming of T-cell responses. In this study, we demonstrated that administration of novel self-amplifying mRNA (SAM^®^) vectors expressing influenza NP (SAM(NP)), M1 (SAM(M1)), and NP and M1 (SAM(M1-NP)) delivered with lipid nanoparticles (LNP) induced robust polyfunctional CD4 T helper 1 cells, while NP-containing SAM also induced cytotoxic CD8 T cells. Robust expansions of central memory (T_CM_) and effector memory (T_EM_) CD4 and CD8 T cells were also measured. An enhanced recruitment of NP-specific cytotoxic CD8 T cells was observed in the lungs of SAM(NP)-immunized mice after influenza infection that paralleled with reduced lung viral titers and pathology, and increased survival after homologous and heterosubtypic influenza challenge. Finally, we demonstrated for the first time that the co-administration of RNA (SAM(M1-NP)) and protein (monovalent inactivated influenza vaccine (MIIV)) was feasible, induced simultaneously NP-, M1- and HA-specific T cells and HA-specific neutralizing antibodies, and enhanced MIIV efficacy against a heterologous challenge. In conclusion, systemic administration of SAM vectors expressing conserved internal influenza antigens induced protective immune responses in mice, supporting the SAM^®^ platform as another promising strategy for the development of broad-spectrum universal influenza vaccines.

## Introduction

Influenza virus is a respiratory pathogen responsible for 250,000–500,000 deaths annually worldwide [1], and vaccination is the most cost-effective way to prevent and control influenza outbreaks [2]. Currently licensed inactivated influenza vaccines (IIV) include the hemagglutinin (HA) viral surface protein, inducing strain-specific antibody responses that protect against antigenically matched or closely related viruses. However, due to the high mutation rate in HA, yearly update of seasonal vaccines is required to match the circulating viruses. In addition, seasonal vaccines are not effective against newly emerging influenza viruses or pandemic outbreaks [3, 4]. For this reason, a “universal” influenza vaccine that could offer broad-range of protection against all subtypes of influenza A virus has been the focus of research efforts for the last two decades. The addition of adjuvants, such as MF59, increases the breadth of immunity elicited by seasonal and pandemic influenza vaccines [5, 6], but not sufficiently to overcome the limitation of seasonal vaccine strain changes [7].

Therefore, several new approaches have been taken towards the development of universal influenza vaccines, including the induction of heterosubtypic immunity directed against internal, antigenically conserved proteins, such as the nucleoprotein (NP) and the matrix protein 1 (M1). These antigens are highly conserved and share over 90% of homology at the amino acid level among the different influenza strains [8, 9]. In addition, T-cell responses to NP or M1 antigens are associated with early virus clearance and reduced disease severity in absence of neutralizing antibodies [10, 11]. NP can elicit cross-reactive cytotoxic T lymphocytes useful to accelerate viral clearance [12] in addition to non-neutralizing antibodies that might have antiviral activity [13]. Previous preclinical studies have demonstrated that different types of vaccines containing NP alone or in combination with other influenza antigens, including plasmid DNA [10, 14–17], double-stranded (ds) DNA viral vectors [18, 19], peptide [20], or adjuvanted protein subunit [21, 22], can protect against homologous and also heterosubtypic influenza challenge.

Vaccination with live attenuated influenza vaccine (LAIV), but not with IIV, was shown to induce T-cell responses against internal influenza antigens [23] that may correlate with the better heterosubtypic protection observed with LAIV than with IIV in children [24]. However, the efficacy of LAIV can be affected by the age of the vaccinees and the extent of antigenic similarity between the vaccine and the circulating strains [25].

Nucleic acid-based vaccines based on mRNA may provide a potent alternative to the previously mentioned approaches. Preclinical studies for both prophylactic and therapeutic mRNA vaccines have demonstrated their ability to elicit functional antibodies and T-cell responses [26–28]. mRNA vaccines preclude safety concerns about DNA integration into the host genome and can be directly translated in the host cell cytoplasm, circumventing the hurdle presented by nuclear transport. Moreover, the simple cell-free, *in vitro* synthesis of RNA avoids the manufacturing complications associated with viral vectors. Two different forms of RNA-based vaccines are currently being developed against influenza: conventional, non-amplifying mRNA [26] and self-amplifying mRNA [29, 30] molecules. Non-viral delivery of self-amplifying mRNAs (SAM^®^ technology), derived from a modified alphavirus single-stranded (ss) RNA genome, elicits very potent and broad-based immune responses due to antigen expression in host cells and to their intrinsic innate immune stimulating capabilities [31, 32]. The SAM^®^ technology has already proven successful against various disease targets like HIV, RSV, CMV, and influenza, and in animal models including mice, cotton rats, ferrets, and non-human primates [27–29, 31].

In the present study and towards the development of a broad-spectrum influenza vaccine, we evaluated in mice the immunogenicity and efficacy of SAM vectors encoding NP and M1 antigens, separately or in combination, using lipid nanoparticles (LNP) as a synthetic, non-viral delivery system [31]. Here we show that SAM vectors are immunogenic, inducing antigen-specific antibody and T-cell responses, and protective in mice against homologous and heterosubtypic influenza virus challenge.

## Materials and Methods

### RNA synthesis

RNA was prepared as previously reported [33]. Briefly, DNA plasmids encoding full-length NP (SAM(NP)) or M1 (SAM(M1)) from the influenza virus A/Puerto Rico/8/34 (H1N1) were amplified in *Escherichia coli* and purified using Qiagen Plasmid Maxi kits (Qiagen). DNA was linearized immediately following the 3’ end of the self-amplifying RNA sequence by digestion with PmeI. Linearized DNA templates were transcribed into RNA using the MEGAscript T7 kit (Life Technologies) and purified by LiCl precipitation. RNA was then capped using the ScriptCap™ m7G Capping System (Cell Script) and purified by LiCl precipitation. In the bicistronic vector (SAM(M1-NP)), both NP and M1 genes were cloned downstream of two distinct 26S subgenomic promoters in order to obtain two separate and entire proteins.

### RNA vector self-amplification and protein expression

Baby hamster kidney cells (BHK, ATCC) were cultured in Dulbecco’s modified Eagle’s medium (Gibco, Carlsbad, CA) containing 5% fetal bovine serum (Hyclone) at 37°C and 5% CO_2_, and used at 90% confluence at the time of transfection.

To determine the efficiency of RNA self-amplification, BHK cells were electroporated (120V, 25ms pulse) with 200 ng of RNA and incubated for 16–18 h at 37°C and 5% CO_2_. Cells were collected, stained with Live/Dead Aqua (Invitrogen), fixed and permeabilized with Cytofix/Cytoperm (BD Biosciences), and stained with APC-conjugated anti-double stranded (ds)RNA antibody (J2 monoclonal mAB mouse IgG2a kappa chain, Bioclass). Anti-dsRNA IgG2a was conjugated using the Zenon Allophycocianin labeling kit (Invitrogen). Frequencies of dsRNA^+^ cells were measured by flow cytometry on a FACS CANTO II flow cytometer (BD Biosciences).

To determine the efficiency of protein expression, BHK cells were transfected with 3 μg of each SAM construct and LIPOFECTAMINE 2000^TM^ (Invitrogen) according to manufacturer’s instructions. BHK cells were collected 18–20 h after transfection, stained with Live/Dead Aqua, then fixed and permeabilized with Cytofix/Cytoperm for flow cytometry analysis. NP expression was assessed using FITC-conjugated anti-NP monoclonal IgG2a (Thermo Fisher Scientific/ Pierce), whereas M1 expression was detected using anti-M1 monoclonal IgG1 (AbD Serotec) and APC-conjugated anti-IgG1 secondary antibody (Invitrogen).

For Western blot analyses, transfected BHK cells were lysed and whole cell lysates of 2x10^5^ cells were subjected to SDS-PAGE and blotted to PVDF membranes. NP was detected with anti-NP HB65 purified hybridoma (1:50) (ATCC Clone H16-R10-4R5), and M1 was detected with anti-M1 monoclonal antibody (1:200) (AbD Serotec), followed by horseradish peroxidase-conjugated goat anti-mouse IgG secondary antibody (1:5000) (PerkinElmer). Protein bands were visualized by chemiluminescence following manufacturer’s instructions (Pierce Protein Research Products, Rockford, IL).

### LNP/RNA formulation

RNAs were formulated with LNPs as previously described [34] and diluted to the desired RNA concentration (1 ng/μl) with PBS. For the SAM(NP)+SAM(M1) formulation, an equal amount of SAM(NP) and SAM(M1) RNAs was mixed prior to complexion with LNP. In this case, the final formulated vaccine was diluted to 2 ng/μl to maintain the same amount of RNA expressing NP and M1 as in the single vectors. Formulations were characterized for particle size, RNA concentration, encapsulation efficiency and antigen expression in transfected cells.

### Influenza virus

Mouse-adapted influenza virus A/PR/8/1934 (H1N1) (PR8) (A. Wack, The Francis Crick Institute, London), influenza virus A/Hong Kong/1/1968 (H3N2) (HK68) (BEI Resources, NIAID, NIH) and A/California/7/2009 (H1N1) (NYMC-X181) reassortant influenza virus (Cal) (Novartis Vaccines S.r.l., Siena) were grown in allantoic cavity of embryonated chicken eggs. Viruses were titrated on Madin-Darby canine kidney (MDCK) cells, quantified as Tissue Culture Infectious Dose that yields 50% infection (TCID_50_), and stored at -80°C. A clinical score system was used to define the mouse lethal doses for PR8 and HK68 viruses. A clinical score ranging from 0 (no symptoms) to 4 (moribund) was ascribed to each mouse and a score of 4 was defined as a humane endpoint.

### Animal studies

Mouse immunogenicity and efficacy studies were conducted at the GSK Vaccines Animal Research Center, in compliance with the ARRIVE guidelines, the current Italian legislation (Legislative Decree 116/92), and with the GSK Animal Welfare Policy and Standards.

BALB/c mice (Charles River Laboratories, Calco, Italy), aged 6–8 weeks, were immunized intramuscularly (i.m.) on days 0 and 56 in the quadriceps muscles of both hind legs (50 μl vaccine formulation per leg) with 0.1 μg of SAM(NP), SAM(M1) or SAM(M1-NP), or 0.2 μg of SAM(NP)+ SAM(M1). In parallel, groups of mice were infected intranasally (i.n.) (15 μl per nostril) with low doses of PR8 (0.025 TCID_50_) or HK68 (1.56 TCID_50_) influenza viruses as controls. For MIIV+SAM co-administration studies, 0.1 μg of MIIV from influenza strain A/California/7/2009 (H1N1) and 0.1 μg of different SAM vaccines were co-formulated prior to injection and administered in the same syringe.

### Determination of NP and M1-specific serum antibody titers by ELISA

NP or M1-specific IgG titers were determined on individual sera collected 3 weeks after the first and 2 weeks after the second immunization. Maxisorp plates (Nunc) were coated overnight at 4°C with 0.26 μg/well of NP or M1 proteins (Sinobiological) and blocked with SmartBlock (Candor) for 1 h at 37°C. Serum samples and a standard serum, 2-fold serially diluted in PBS, 1% BSA, 0.05% Tween 20, were transferred into coated and blocked plates and then incubated 1 h at 37°C. To detect antigen-specific IgG antibodies, plates were incubated with alkaline phosphatase-conjugated goat anti-mouse IgG (Sigma) for 90 min at 37°C. Then the P-nitrophenyl phosphate disodium substrate was added and the reaction was stopped by adding 3% EDTA pH 8. Absorbance was measured with a SpectraMax reader (Molecular Devices) at 405 nm. The titers were normalized with respect to the reference serum assayed in parallel and are indicated as ELISA Units/ml (EU/ml).

### Virus neutralization assay

Heat-inactivated pooled sera were 3-fold serially diluted in minimal essential medium (Gibco) with Penicillin, Streptomycin, Glutamine 100X Solution (Life Technologies), Trypsin 1X 1:250, and incubated 1 h at 37°C with 100 TCID_50_ of influenza Cal virus or with 300 TCID_50_ of PR8 virus, equivalent working dilutions for the ELISA-based microneutralization assay, as confirmed by the virus back titration included in each experimental plate (data not shown). The first dilution tested was 1:80. All samples were then incubated for 18 h at 37°C and 5% CO_2_ on MDCK cells, plated in a 96-well plate (2x10^4^ cells/well). Cells were then washed with PBS, fixed with Fixation Buffer (BD Cytofix) and permeabilized with a solution of PBS 0.1% BSA 0.1% Tween 20. Plates were incubated with FITC-conjugated monoclonal antibody against M1 and NP antigens (α-M/NP-FITC) (Oxoid) for 1 h and then washed and incubated with horseradish peroxidase-conjugated anti-FITC polyclonal antibody (Roche). O-phenylenediamine dihydrochloride (Sigma) was used as substrate and the absorbance recorded at 450 nm using a SpectraMax reader (Molecular Devices). Inhibition of infection of 50% was determined by a 4-parameters fitting curve (SoftMaxPro) and the corresponding titers represented as the reciprocal of the dilution. A titer of 40 was assigned to sera that gave a negative result at the first dilution tested (1:80).

### Intracellular cytokine staining (ICS)

To assess antigen-specific T-cell responses, single-cell suspensions were prepared from spleens or lungs, and 10^6^ cells were plated with anti-CD28 mAb at a final concentration of 2 μg/ml (Pharmingen) and with anti-CD107a FITC (2.5 μg/ml; BD Biosciences).

Cells were stimulated for 6 h with H-2K^d^-restricted NP peptide TYQRTRALV (2.5 μg/ml; JPT), recombinant NP protein (5 μg/ml; Sino Biological Inc.), or with a M1 peptide pool library consisting 15-mer peptides overlapping by 10 amino acids (2,5 μg/ml; Department of Biochemistry, University of Lausanne, Switzerland). For H1-specific T-cell responses, a Cal/H1 peptide pool (JPT) or a PR8/H1 peptide pool (Department of Biochemistry, University of Lausanne, Switzerland) were used for *in vitro* stimulation. The same number of cells was incubated with anti-CD3 and anti-CD28 (2 μg/ml each) or anti-CD28 alone as positive and negative controls, respectively. Brefeldin A (5 μg/ml; Sigma) was added for the last 4 h.

For flow cytometry analyses, cells were then stained with Live/Dead Near InfraRed (Invitrogen), anti-CD62L A700 (BD Pharmingen), and anti-CD127 APC (eBioscience), fixed and permeabilized with Cytofix/Cytoperm (BD Biosciences), and then incubated with anti-CD16/CD32 Fc block (BD Biosciences). T cells were further stained with anti-CD3 PerCP-Cy5.5, anti–CD4 V500, anti–IFN-γ Brilliant Violet 785, anti–IL-2 PE-Cy5.5, anti–TNF Brilliant Violet 605, and anti–CD44 V450 (all from BD Pharmingen), anti-IL-4 PE and anti-IL-13 PE (from eBioscience), and anti-CD8 PE Texas Red (Invitrogen). Samples were acquired on a LRSII special order (BD Biosciences), and analyzed using FlowJo software version 9.7.4 (TreeStar). T cells were identified as previously described [30], and frequencies of antigen-specific T cells were calculated after subtracting the background measured in the corresponding negative control for each cytokine.

### *In vivo* cytotoxicity assay

BALB/c mice were immunized i.m. twice 8 weeks apart with 0.1 μg of SAM(NP), 0.2 μg of SAM(NP)+SAM(M1) or 0.1 μg of SAM(M1-NP). To prepare target cells, splenocytes from naïve mice were split into two populations. One population was pulsed for 1 h at 37°C with 5 μM of NP_147-155_ peptide (TYQRTRALV), washed, and labeled with 0.5 μM of CFSE (CFSE^+^ cells). The other population was pulsed with unrelated HIV_197-205_ peptide (AMQMLKETI), and labeled with 10 μM of CMTMR (CMTMR^+^ cells). An equal number of cells (5x10^6^ total splenocytes) from the two different peptide-pulsed and labeled populations was mixed and injected i.v. into immunized mice 10 days after the second immunization. Mice were sacrificed 18 h later and splenocytes were analyzed on a LRSII special order (BD Biosciences) to determine the frequencies of CFSE^+^ and CMTMR^+^ cells.

The specific lysis was calculated as previously reported [35]:

$$R=\frac{\%\;CMTMR+cells}{\%CFSE+cells}$$

$$Specific\;Lysis\;(\%)=[1-\left(\frac{Rc}{Rimm}\right)]\times 100$$

where Rc is the ratio of PBS control mice and Rimm is the ratio of immunized mice

### Influenza virus challenge

Four weeks following the last immunization, anesthetized mice were challenged i.n. with equivalent lethal doses of PR8 (10.5 TCID_50_) or HK68 (1560 TCID_50_) mouse-adapted influenza viruses (15 μl per nostril). Survival, body weight, and clinical signs of illness (e.g. ruffled fur, hunched posture, wheeze) were monitored daily for 2 weeks after infection. A clinical score of 4 was defined as a humane endpoint and animals meeting this criterion were euthanized. For MIIV+SAM co-administration studies, mice were infected with 105 TCID_50_ of PR8.

### Lung influenza viral load

Whole mouse lungs were collected in Hank's Balanced Salt Solution and homogenized using a Gentle MACS dissociator (Miltenyi). An aliquot was collected, centrifuged at 320 g for 10 min, and suspended in Trizol (Life Technologies). Total RNA was extracted using phenol/chloroform, and cDNAs were generated using the Thermoscript RT-PCR system following the manufacturer’s instructions (Life Technologies). The cDNA served as a template for the amplification of influenza M1 gene and eukaryotic HPRT1 housekeeping gene by real-time PCR. Quantitative RT-PCR was performed in triplicate for each cDNA sample using the TaqManUniversal Master Mix with UNG (Applied Biosystems).

Forward primer: 5-AAGACCAATCCTGTCACCTCTGA-3; reverse primer: 5-CAAGCG TCTACGCTGCAGTCC-3; and probe: 5-[(6FAM)TTTGTGTTCACGCTCACCGT (TAM)]-3 were used to amplify the influenza M1 gene. The thermal cycling program was as follows: an initial 2 min at 50°C and 10 min at 95°C, followed by 40 cycles of 15 s at 95°C and 1 min at 60°C. RT-PCR was analyzed using the LightCycler 480 System (Roche).

The viral titers were calculated using the comparative C_t_ method [36] and were indicated as fold-increase to pre-infected sample. M1 mRNA expression was normalized to levels of HPRT1 mRNA expressed in the corresponding sample.

### Lung processing and characterization of T cells

To assess viral titers, cell recruitment and T-cell responses induced by PR8 infection, lungs were excised at 0, 3, 6 and 17 days after challenge. Lung tissue was completely dissociated with Gentlemax Dissociator (Milteny Biotec), as previously described [37]. Briefly, lung tissue was digested in Hank's Balanced Salt Solution (Life Technologies) containing calcium and magnesium in presence of collagenase D (2 mg/ml) and DNAse I (80 units/ml) (Roche) for 30 min at 37°C, and then homogenized until obtaining a single-cell suspension.

To characterize NP-specific CD8^+^ T cells recruited in the lungs, 10^6^ cells were stained with PE-labeled NP_147-155_-specific H-2K^d^ pentamer (Proimmune) for 20 min in PBS 2% fetal bovine serum, and then with Live/Dead Fixable Yellow and anti-CD8 PE Texas Red (Invitrogen), anti-CD3 PerCP-Cy5.5, anti-CD4 V500, anti-CD44 V450 and anti-CD19 FITC. ICS of antigen-specific CD8^+^ and CD4^+^ T cells was performed as previously described. Samples were analyzed on a FACS LSR II Special Order System (BD Biosciences), using BD DIVA software (BD Biosciences).

### Ethics statement

All animal studies were carried out in compliance with the ARRIVE guidelines, the current Italian legislation on the care and use of animals in experimentation (Legislative Decree 116/92), and with the GSK Animal Welfare Policy and Standards. Protocols were approved by the Italian Ministry of Health (authorization 249/2011-B and 22/2015-PR), and the Novartis Vaccines Animal Welfare Body (authorizations AWB 201106 and 201522). Following infection, mice were monitored daily and euthanized when they exhibited defined humane endpoints that were pre-established in agreement with GSK Animal Welfare Policies.

### Statistical analysis

Statistical analyses were performed using GraphPad Prism 6.04 software. Experiments involving animal survival were analyzed by Mantle-Cox Log-rank test. For the other statistical analyses, Mann-Whitney U test was used, unless otherwise indicated. P values less than 0.05 were considered statistically significant.

## Results

### Generation and characterization of SAM encoding influenza NP and M1 antigens

**Fig 1:**
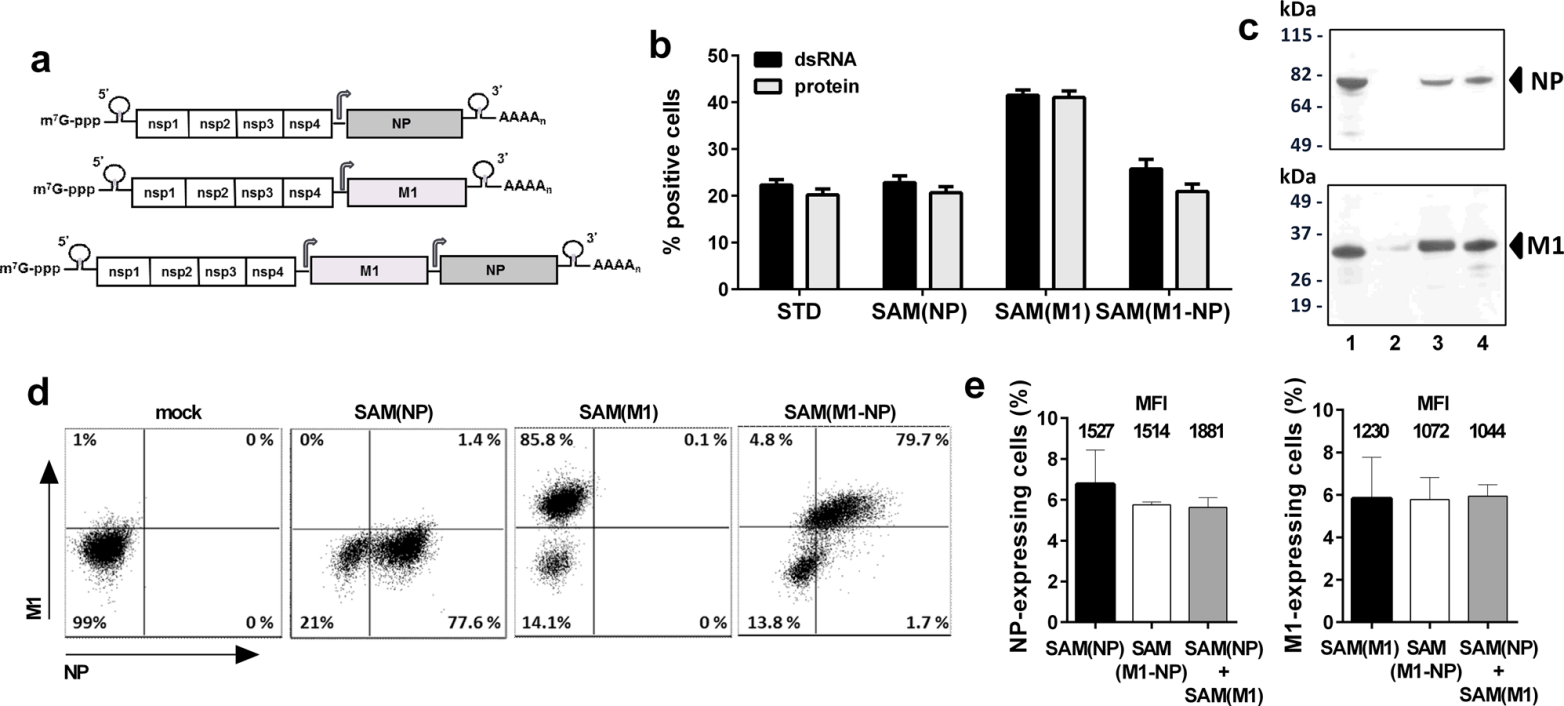
Schematic representation of SAM vectors and their characterization *in vitro*. (a) SAM(NP), SAM(M1) and SAM(M1-NP) constructs showing a 5’ cap, four non-structural genes (nsp1-4), a 26S subgenomic promoter (grey arrow), the vaccine antigen(s), and a 3’ polyadenylated tail. (b-d) Self-amplification of SAM replicons and antigen expression assessed after transfection of BHK cells with the different replicons. (b) Percentage of BHK cells positive for replicating SAM vectors (dsRNA^+^ cells) and expressing the corresponding protein (protein^+^ cells) was analyzed by flow cytometry and indicated as mean ± SD. (c) Cell lysates from BHK cells infected with the PR8 virus (0.1 multiplicity of infection) (lane 1), mock-transfected (lane 2), or transfected with SAM(NP) (top panel) and SAM(M1) (lower panel) (lane 3), or SAM(M1-NP) (lane 4) were analyzed by Western blot under reducing conditions. (d) Frequency of NP- and M1-expressing BHK cells transfected with mock, SAM(NP), SAM(M1) or with the bicistronic SAM(M1-NP) replicons were analyzed by flow cytometry. (e) Frequency (mean ± SD) and mean fluorescence intensity (MFI) of BHK cells expressing NP or M1 antigens after transfection with the different SAM/LNP formulations. Statistical analyses were performed using the Mann-Whitney U test. *p<0.05 compared to the SAM(NP)- or SAM(M1)-treated groups. Data shown are representative of three independent experiments.

The full-length NP and M1 genes were amplified from the reverse-transcribed RNA genome of influenza virus A/PR/8/34 (H1N1), and then cloned into the DNA plasmid backbone as two monocistronic (SAM(NP) or SAM(M1)), and one bicistronic (SAM(M1-NP)) vectors (Fig 1A). The corresponding ssRNAs were synthesized *in vitro* by an enzymatic transcription reaction from a linear plasmid DNA template using a T7 RNA polymerase [31]. The *in vitro* activity of the monocistronic and bicistronic SAM replicons was measured after electroporation in BHK cells and compared to a control self-amplifying RNA of known potency (STD). The presence of intracellular dsRNA molecules, as markers of RNA amplification, was evaluated by flow cytometry (Fig 1B). The frequencies of dsRNA^+^ BHK cells after transfection with the two SAM(NP) and SAM(M1) monocistronic or the SAM(M1-NP) bicistronic replicons were comparable or higher than that obtained with the STD, indicating that the new replicons self-amplified. The frequencies of dsRNA^+^ and protein^+^ cells were comparable for each replicon, suggesting that antigen expression paralleled RNA amplification.

Antigen expression by BHK cells after transfection with the different SAM replicons was further characterized by Western Blot (Fig 1C) and flow cytometry (Fig 1D). Both M1 and NP proteins expressed by the monocistronic (lane 3) or bicistronic (lane 4) vectors showed bands in the western blots with molecular weights equivalent to the respective proteins expressed by PR8 virus-infected BHK cells (lane 1) (Fig 1C). Finally, the percentage of NP or M1 expressing BHK cells was greater than 70% for monocistronic and bicistronic replicons, and the majority of BHK cells transfected with the bicistronic replicon co-expressed M1 and NP (Fig 1D). For *in vivo* studies, SAM vectors were encapsulated with LNPs. Mean particle size and polydispersity were measured by dynamic light scattering for SAM(NP)/LNP, SAM(M1)/LNP, [SAM(M1)+ SAM(NP)]/LNP, and SAM(M1-NP)/LNP. Z-average diameters ranged from 130 to 142 nm with a low polydispersity index (data not shown), indicating small uniform lipid particles able to encapsulate more than 95% of the mRNA [31]. Finally, flow cytometry analysis showed that the percentage and MFI of BHK cells expressing the NP or M1 antigens after transfection with the different SAM/LNP formulations were comparable between single antigen and combination groups (Fig 1E).

### Immunogenicity of SAM(NP) and SAM(M1) vaccines

To assess the immunogenicity of SAM replicons expressing NP and/or M1 antigens, BALB/c mice were immunized i.m. twice, eight weeks apart, with 0.1 μg of SAM(NP), SAM(M1), a mixture of both SAM(NP)+SAM(M1), or SAM(M1-NP) and delivered with LNP. Infection with a low dose of the PR8 virus and treatment with PBS were used as positive and negative controls, respectively.

NP- and M1-specific IgG were already detectable in the sera of SAM-immunized mice after the first dose and were boosted by the second immunization (S1A Fig). Mice that had received the single antigens reached antibody titers 1.5–2 -fold higher (*p*<0.01) than those vaccinated with both antigens, suggesting that NP and M1 induce mild antigenic interference [38, 39]. Sera from SAM-immunized mice failed to neutralize PR8 virus infection of MDCK cells *in vitro* (S1B Fig), consistent with the internal location of these antigens in the virus [40].

**Fig 2:**
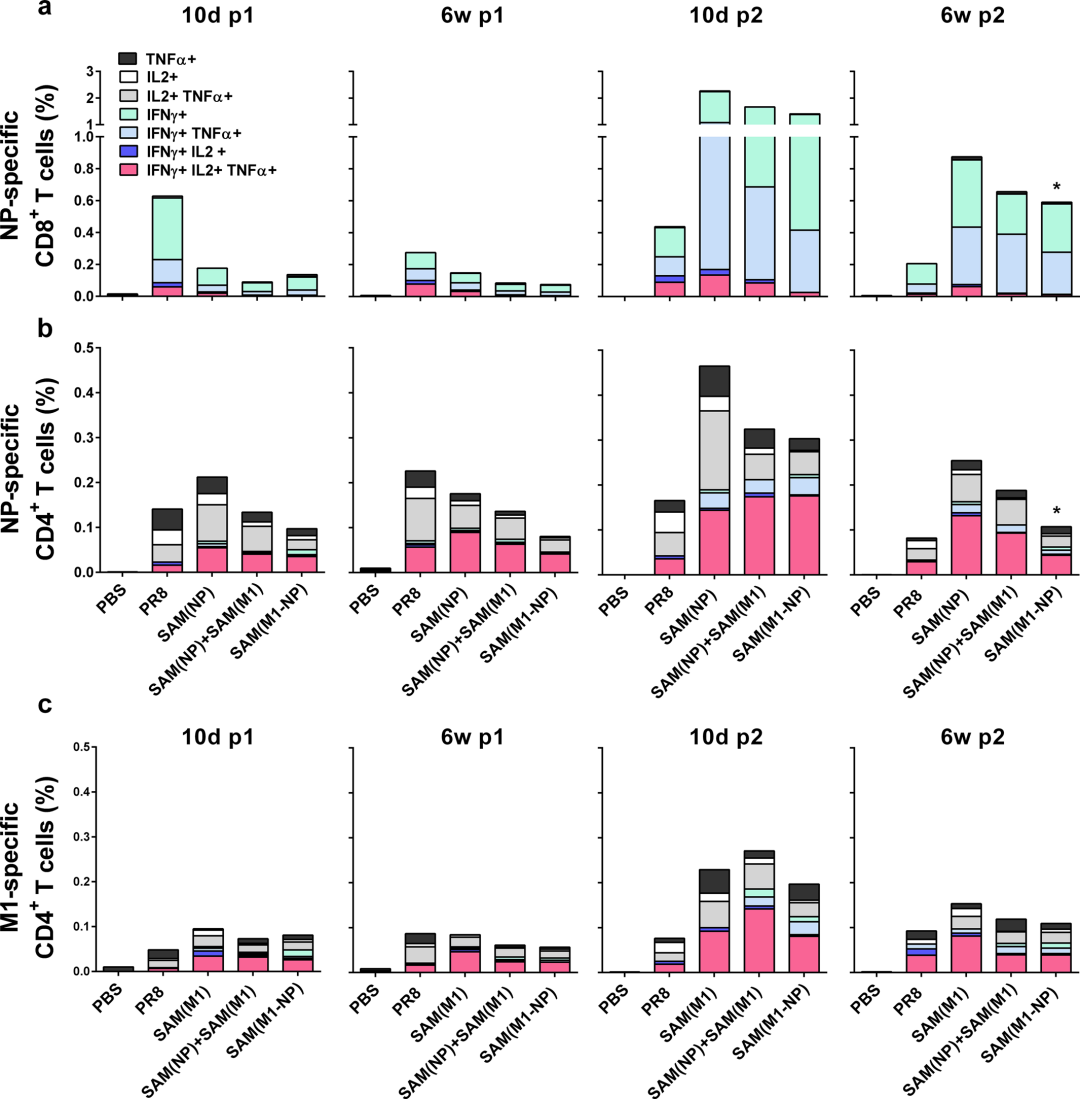
T-cell responses induced by SAM formulations. (a-b) BALB/c mice (n = 24/group) were immunized i.m. twice, 8 weeks apart, with 0.1 μg of SAM(NP), SAM(M1), SAM(M1-NP), or with 0.2 μg of SAM(NP)+SAM(M1). Ten days and 6 weeks after each immunization, the frequency of antigen (Ag)-specific, cytokine-secreting CD8^+^ (a) or CD4^+^ (b, c) T cells was determined by flow cytometry on splenocytes stimulated *in vitro* with the NP_147-155_ peptide (a), recombinant NP protein (b) or with the M1-derived peptide pool (c). Color code indicates the different combinations of cytokine produced by the respective cells. As control, a group of mice were infected with a low dose of influenza virus PR8. Data derived from two separate and merged experiments. Statistical analysis was performed using the Mann-Whitney U test. *p<0.05 compared to SAM(NP) (a-b) and SAM(M1) (c). Frequencies of Ag-specific CD4^+^ and CD8^+^ T cells were significantly higher (*p*<0.05) in all SAM-immunized and PR8-exposed groups than in PBS at all time points. Induction of antigen-specific memory T cells by SAM(NP) and SAM(M1) vaccines

Based on the key protective role played by NP- and M1-specific T cells against influenza disease [12, 14], we focused the next set of analyses on the characterization of antigen-specific T-cells by ICS and flow cytometry (Fig 2). Antigen-specific, cytokine-secreting cells were identified among the CD44^high^ CD8^+^ and CD4^+^ T cell subsets as previously described [30] in splenocytes of immunized animals stimulated *in vitro* with NP_147-155_ peptide (Fig 2A), recombinant NP protein (Fig 2B) or with M1-derived peptide pool (Fig 2C). NP-specific CD8^+^ T cells were already detectable 10 days after the first immunization and maintained at frequencies around 0.1–0.2% until week 6 (Fig 2A). A 10-fold increase was seen 10 days after the second immunization, with frequencies of NP-specific CD8^+^ T cells ranging from 1 to 2% of total CD8^+^ T cells and contracting to 0.6–0.9% at 6 weeks. The majority of NP-specific CD8^+^ T cells were IFN-γ^+^ and IFN-γ^+^/TNF-α^+^, characteristic of an effector phenotype. No M1-specific CD8^+^ T-cells were detected in PR8-exposed mice and in any SAM(M1) vaccine group at any time point tested (data not shown).

Similarly, NP-specific CD4^+^ T cells were already detectable 10 days after the first immunization, and were predominantly Th0 (IL-2^+^/TNF-α^+^, TNF-α^+^, and IL-2^+^) and multifunctional Th1 (IFN-γ^+^/IL-2^+^/TNF-α^+^) (Fig 2B). The second immunization expanded NP-specific CD4^+^ T cells in all the immunization groups, especially the multifunctional Th1 sub-population (IFN-γ^+^/IL-2^+^/TNF-α^+^). Frequencies of NP-specific CD4^+^ T cells ranged from 0.1–0.2% up to 6 weeks after the first immunization, increased up to 0.3–0.5% 10 days after the second immunization, and decreased to 0.1–0.3% at 6 weeks thereafter. No significant differences in terms of intensity or quality of the responses were observed between the different SAM vaccinated groups except for the 6 weeks post 2 time point when the intensity of NP-specific T cell responses were significantly reduced in mice immunized with SAM(M1-NP) compared to the other two SAM-vaccinated groups. M1-specific CD4^+^ T-cells showed kinetics and phenotype similar to NP-specific CD4^+^ T cells, albeit with lower frequencies (Fig 2C). No differences were observed in the frequencies of antigen-specific T-cells induced by SAM(M1) alone or in combination with SAM(NP).

Mice pre-exposed to a low dose of the PR8 virus showed approximately 0.6% and 0.1% of NP-specific CD8^+^ and CD4^+^ T cells, respectively, that did not increase after a second exposure to the virus. It is likely that HA-specific antibodies induced by the first exposure to PR8 neutralized the second virus infection, thus preventing the recall and expansion of NP-specific T-cells. Low frequencies of M1-specific CD4^+^ T-cell responses, but no M1-specific CD8^+^ T cells were detected in infected mice, as was observed for SAM immunized mice. No NP- or M1-specific T cells were detected in PBS-treated mice. Similar immune profiles were observed when total numbers, rather than frequencies, of antigen-specific CD4 and CD8 T cells were reported (data not shown).

**Fig 3:**
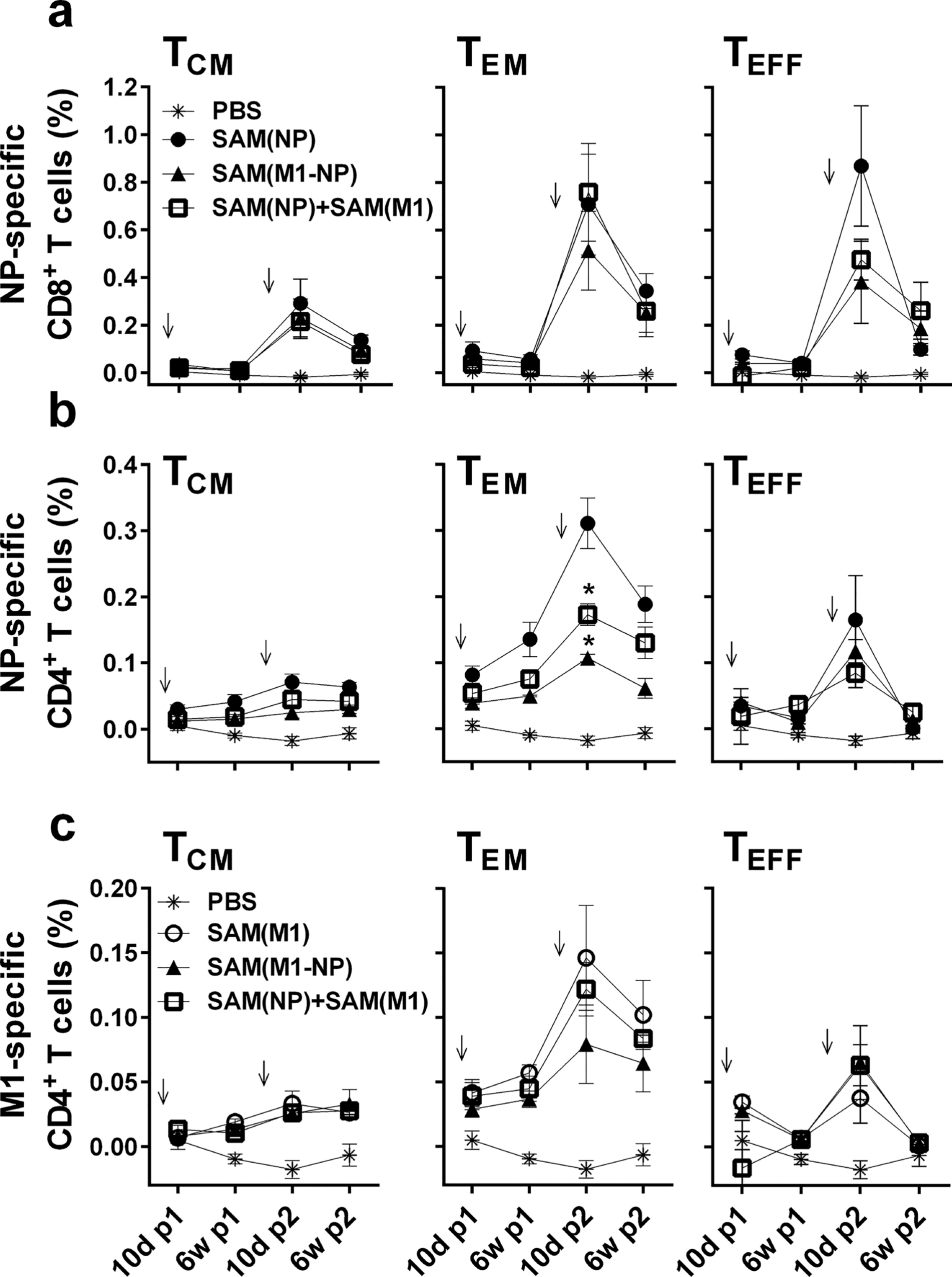
SAM vaccines induce Ag-specific effector and central memory T cells. Ten days and 6 weeks after each immunization, the frequency of NP-specific (a-b) or M1-specifc (c) cytokine-secreting cells were determined within the central memory: T_CM_ (CD44^high^/CD62L^high^/CD127^high^), the effector memory: T_EM_ (CD44^high^/CD62L^low^/ CD127^high^), and the effector: T_EFF_ (CD44^high^/CD62L^low^/CD127^low^) subsets. Arrows indicate the immunization times. Data derived from two separate and merged experiments. Statistical analyses were performed using the Mann-Whitney U test. *p<0.05 compared to SAM(NP) (a-b) or SAM(M1) (c).

In addition to cytokine secretion, we characterized the memory phenotype of antigen-specific T cells in spleens of immunized mice by measuring the frequency of effector (T_EFF_, CD44^high^/CD62L^low^/CD127^low^), effector memory (T_EM_, CD44^high^/CD62L^low^/ CD127^high^), and central memory (T_CM_, CD44^high^/CD62L^high^/CD127^high^) T cells at different time points upon vaccination (Fig 3).

Low frequencies of antigen-specific T_CM,_ T_EM_ and T_EFF_ cells were measured after the first immunization with all SAM vaccines, while after the second immunization, the frequency of NP-specific CD8^+^ T_CM_ cells was boosted up to 0.2% (Fig 3A), and some NP- and M1-specific CD4^+^ T_CM_ cells were also detected (Fig 3B and 3C). The frequencies of CD8^+^ and CD4^+^ T_EM_ and T_EFF_ increased after the second vaccination, peaking at day 10 and contracting after 6 weeks. This kinetic was observed for both CD8^+^ and CD4^+^, with no major differences among the immunized groups, except for NP-specific CD4^+^ T_EM_ cells which showed a significantly reduced frequency in SAM(NP)+SAM(M1) and SAM(M1-NP) vaccine groups compared to SAM(NP) immunized mice. Altogether, these results suggest that SAM vaccines induce a strong activation of CD8^+^ than CD4^+^ T cells, and expansion of the effector memory compartment.

### Induction of cytotoxic T cells by SAM(NP) vaccine

**Fig 4:**
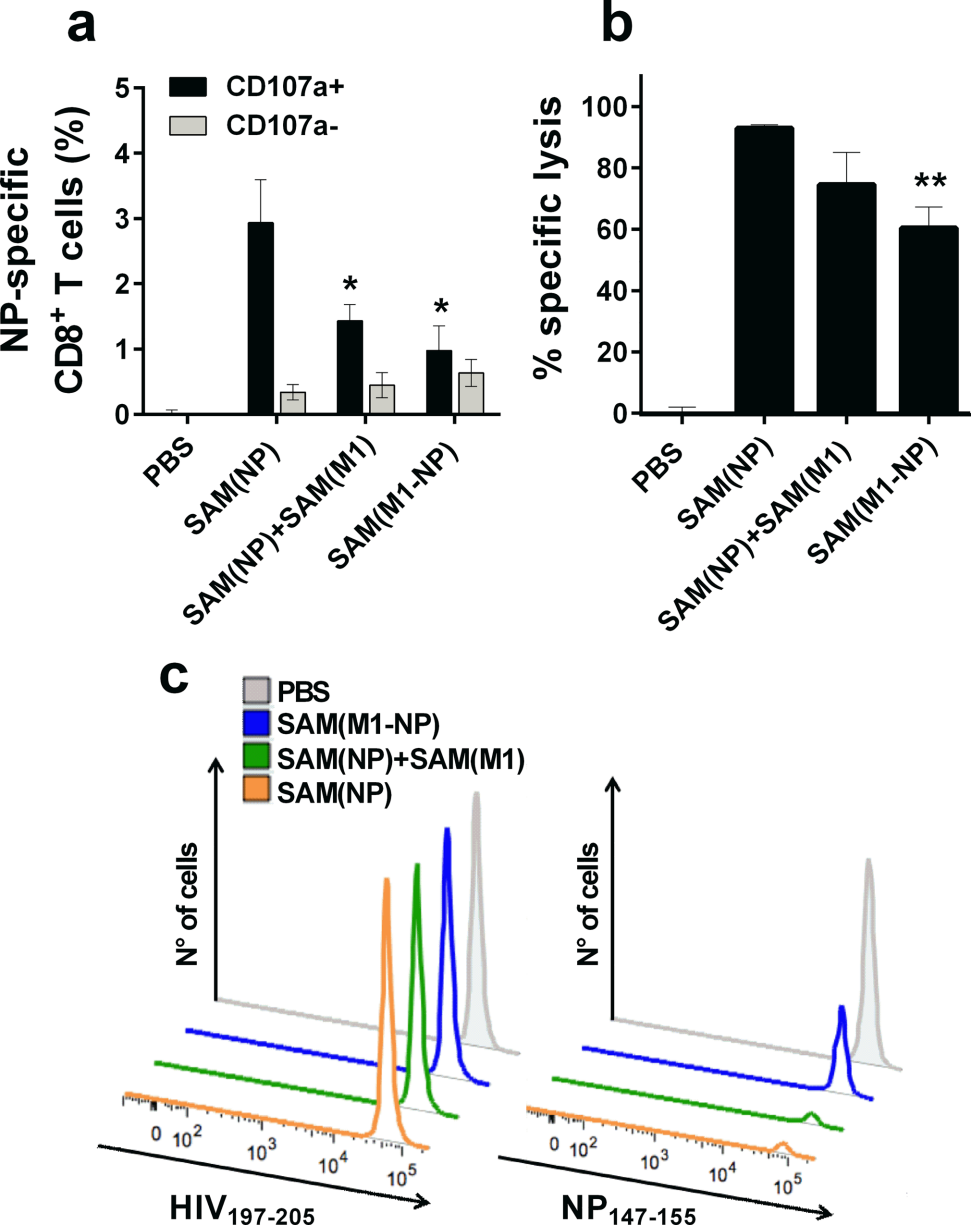
SAM vaccines elicit cytotoxic CD8 T cells. The induction of NP-specific CD8^+^ T cells by SAM(NP) alone or in combination with SAM(M1) was characterized 10 days after the second immunization. (a) Surface expression of CD107a on splenocytes stimulated *in vitro* with NP_147-155_ was assessed by flow cytometry. Data show the frequency of cytokine-secreting CD8^+^ T cells that express (black bars) or not (grey bars) CD107a. (b) Percentage of *in vivo* NP-specific target cell lysis calculated for each immunization group. (c) Representative histograms showing the frequency of influenza NP_147-155_-pulsed (CFSE^+^) and HIV Gag_197-2015_-pulsed (CMTMR^+^) target cells recovered in each immunization group 18 h after adoptive transfer. Statistical analyses were performed using the Mann-Whitney U test. *p<0.05; **p<0.01 compared to SAM(NP).

Finally, we characterized antigen-specific T cells induced by SAM vaccines for cytotoxic activity *in vitro* and *in vivo* (Fig 4). T cell cytotoxicity was evaluated by quantifying the surface expression of CD107a, as a measure of the degranulation process [41], upon *in vitro* antigen-stimulation of splenocytes from immunized animals. After two immunizations with SAM(NP) alone or in combination with SAM(M1), the majority of NP-specific CD8^+^ T cells were CD107a^+^ (Fig 4A). The immunization with SAM(NP) alone induced higher frequency of CD107a^+^ NP-specific CD8 T cells compared to the combination vaccines (*p*<0.05). We did not detect CD107a on NP- or M1-specific CD4^+^ T cells, suggesting that SAM formulations did not induce cytotoxic CD4^+^ T cells.

To evaluate the *in vivo* cytotoxic activity of NP-specific CD8^+^ T cells, an equivalent number of CFSE-labeled or CMTMR-labeled splenocytes were pulsed with the H2-K^d^ -restricted NP_147-155_ peptide (0.5 μM CFSE) or with an unrelated HIV-Gag_197-205_ peptide (10 μM CMTMR), respectively, and were adoptively transferred in mice immunized with 0.1 μg of SAM(NP), 0.2 μg of SAM(NP)+SAM(M1) or 0.1 μg of SAM(M1-NP). The percentage of CFSE^+^ and CMTMR^+^ cells present in the spleens were measured by flow cytometry 18 h later (Fig 4B and 4C). A specific lysis of > 93% was measured in SAM(NP)-immunized mice, while 74% and 60% of specific lysis were detected in SAM(NP)+SAM(M1) and SAM(M1-NP) immunized groups, respectively. No specific lysis was detected in PBS treated mice confirming the antigen-specificity of the cytotoxic activity. These results demonstrated that the SAM formulations induced NP-specific CD8^+^ T cells with cytotoxic activity *in vivo* against target cells pulsed with the H2-K^d^-restricted immunodominant NP peptide. Furthermore, we observed an enhanced *in vivo* cytotoxic activity in SAM(NP) vaccinated mice compared to mice immunized with the combination vaccines, in agreement with the frequencies of CD107a^+^ NP-specific CD8^+^ T cells observed *in vitro* in the respective immunization groups (Fig 4A).

### Protective efficacy in mice against challenge with homologous and heterosubtypic influenza viruses

**Fig 5:**
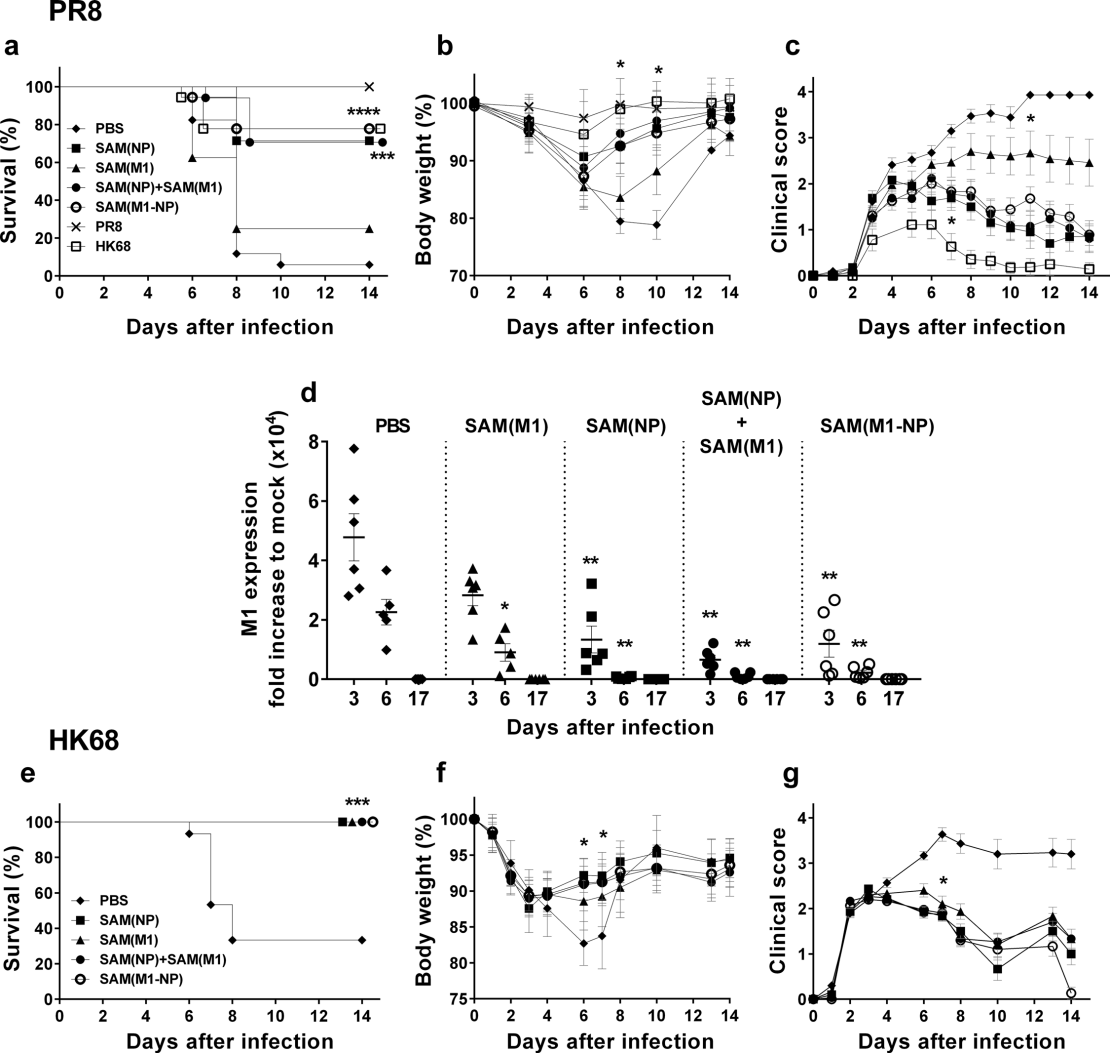
SAM vaccines protect mice against lethal homologous and heterosubtypic influenza challenge. BALB/c mice (n = 18) were immunized i.m. twice, 8 weeks apart, with 0.1 μg of SAM(NP), SAM(M1), SAM(M1-NP), or with 0.2 μg of SAM(NP)+SAM(M1). Four weeks after the last injection, mice were challenged with the homologous PR8 (a-d) or the heterosubtypic HK68 (e-g) influenza viruses. Mice were monitored for survival (a and e) body weight loss (b and f) and clinical score (c and g) for 14 days after infection and euthanized when the clinical score reached 4. Data shown are mean ± SD. (d) Viral titers measured in lungs collected at day 3, 6 and 17 after influenza challenge and expressed as fold-increase compared to pre-infected samples. Individual mice, mean and SD are reported. Data are derived from two independent and merged experiments. Statistical analyses were performed using the Log rank analysis (Mantel Cox test) (a, e), and the Mann-Whitney U test (b, c, d, f, g): *p<0.05, **p<0.01, ***p< 0.001 compared to the PBS-treated group.

To explore the protective efficacy of SAM vaccines, BALB/c mice were immunized twice, eight weeks apart, with 0.1 μg of SAM(NP), SAM(M1), SAM(M1-NP), or 0.2 μg of SAM(NP)+SAM(M1) vectors formulated in LNPs, and challenged with a lethal dose of the mouse-adapted homologous PR8 influenza virus. As controls, we included two groups of mice previously exposed to a low dose of PR8 or HK68 influenza viruses. Survival, weight loss and clinical scores were measured for 14 days after challenge (Fig 5).

Mice immunized with SAM replicons expressing NP, either alone or in combination with M1, and formulated with LNP were protected against lethal infection with the homologous PR8 virus, showing a substantially and significantly higher (*p*<0.0001) survival rate compared to PBS-treated control mice (Fig 5A). Mice immunized with SAM(NP), SAM(NP)+SAM(M1) and SAM(M1-NP) vaccines had survival rates of 71, 70, and 78%, respectively. In contrast, administration of the SAM(M1) vaccine alone was poorly protective (25% survival rate) and not statistically different from PBS-treated control mice (5% survival rate). As expected, PR8 pre-exposed mice were completely protected from the homologous challenge, while mice pre-exposed to heterosubtypic HK68 virus were partially protected (78%), with survival rates similar to those conferred by the SAM(M1-NP) vaccine. Transient body weight loss, as a sign of influenza disease, was observed in all the immunization groups. However, mice vaccinated with the NP-expressing replicons showed a more rapid recovery from disease compared to PBS-treated animals, as demonstrated by significantly reduced body weight loss (Fig 5B) and clinical scores (Fig 5C). Indeed, 3 days after influenza infection, animals immunized with SAM(NP) and combination (NP+M1 or M1-NP) vaccines had significantly reduced lung viral titers compared to PBS-treated mice (*p*<0.01), and completely cleared influenza viral particles from the lungs after 6 days (Fig 5D). Body weight loss and clinical scores indicated an intermediate grade of illness for SAM(M1)-immunized mice, in agreement with the survival results and the slower control of lung viral titers.

To determine the cross-protection conferred by SAM vaccines, we challenged mice with the heterosubtypic HK68 strain and assessed survival, loss in body weight, and overall clinical scores. All SAM vaccines were associated with 100% survival, reduced overall weight loss, and low clinical scores (Fig 5E–5G). In contrast, PBS-treated mice showed a poorer outcome with a 30% survival rate, over 15% body weight loss at the peak of the infection (days 6–8), and clinical scores above 3. The different protective efficacy of SAM(M1) vaccine in the homologous and heterosubtypic infection models could be due to the difference in virulence of PR8 and HK68 viruses [42], as suggested by the different survival rates observed in PBS-treated mice.

### Effect of SAM vaccines on lung T-cell responses after influenza virus challenge

**Fig 6:**
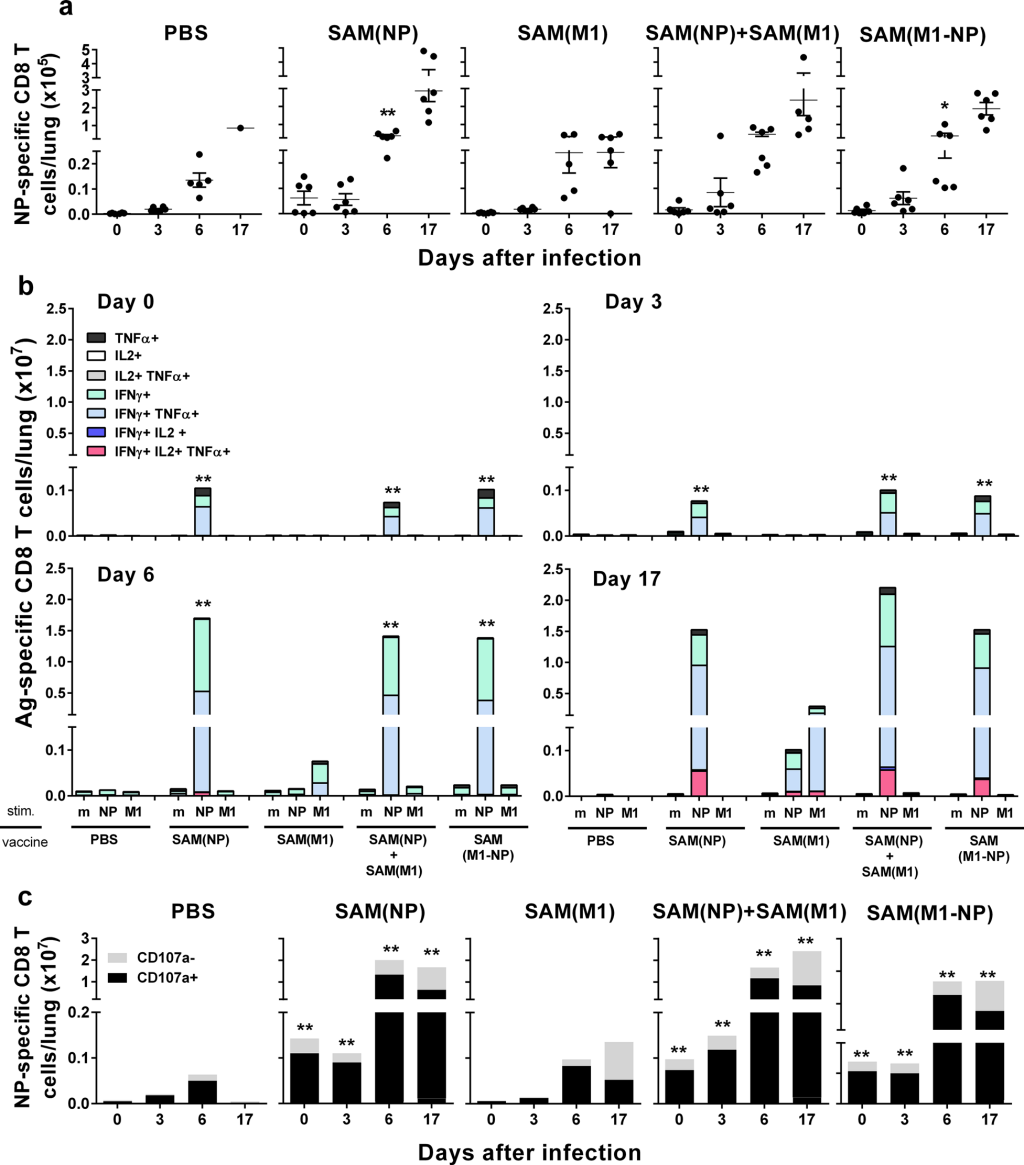
NP-specific CD8^+^ T-cell responses in lungs after influenza challenge. BALB/c mice were immunized i.m. twice, 8 weeks apart, with PBS, 0.1 μg of SAM(NP), SAM(M1), SAM(M1-NP), or with 0.2 μg of SAM(NP)+SAM(M1). Four weeks after the second immunization, mice were infected with PR8 virus. NP-specific CD8 T cells recruited in the lungs after the infection were characterized by flow cytometry. (a) Numbers of NP-specific CD8^+^ T cells. Data are from individual mice (depicted as dots), while solid lanes indicate the mean±SD. (b) Cumulative frequency of Ag-specific, cytokine-secreting CD8^+^ T cells, indicated as absolute number per lung. The color code represents the different combinations of cytokine produced by the respective cells after *in vitro* stimulation with medium (m), NP_147-155_ peptide (NP), or M1 peptide pool (M1), as indicated. (c) Absolute number of NP-specific CD8^+^ T cells positive (black bar) or not (grey bar) for CD107a. Data derived from two independent and merged experiments. Statistical analyses were performed using the Mann-Whitney U test. *p<0.05; **p<0.01 compared to the PBS-treated group.

Vaccination might influence T-cell responses to influenza infection at the site of virus entry. Therefore, we characterized the lung T-cell composition on day 0, 3, 6 and 17 after PR8 challenge in SAM(NP), SAM(M1), SAM(NP)+SAM(M1) or SAM(M1-NP)-vaccinated mice (Fig 6).

At the time of influenza challenge (day 0), H2-K^d^/NP_147-155_ pentamer^+^ CD8^+^ T cells were already detectable in the lungs of mice immunized with SAM(NP) and combinations, but not with SAM(M1) or PBS. Their number increased in all immunization groups at day 6 after infection, and remained high at day 17 in SAM(NP) and combination groups (Fig 6A). IFN-γ^+^/TNF-α^+^, TNF-α^+^, and IFN-γ^+^ NP-specific CD8^+^ T cells were detected in SAM(NP) and combination groups already at day 0. Their frequency increased at day 6 after infection, and showed a more complex IFN-γ^+^/IL-2^+^/TNF-α^+^ phenotype at day 17. Finally, M1-specific CD8^+^ T cells were detected at day 6 and 17 in the SAM(M1) vaccine group, but not in the combination formulation groups (Fig 6B). In agreement with the effector phenotype observed by ICS, most NP-specific CD8^+^ T cells found in the lungs of NP-immunized animals were CD107a^+^ (Fig 6C).

Since antigen-specific CD4^+^ T cells can also have a role in mediating protection by contributing to the development of the effector functions of CD8^+^ T cells [43, 44], we characterized lung NP- and M1-specific CD4^+^ T cells after vaccination and subsequent influenza infection. The characteristic CD4 Th1 profile observed after systemic immunization (Fig 2) was maintained after infection by lung-infiltrating antigen-specific CD4^+^ T cells that expressed IFN-γ and TNF-α, alone or in combination (S2 Fig). Finally, histological analysis of the lungs of vaccinated mice showed that, the high number of effector CD8^+^ and CD4^+^ T cells present from day 6 onwards after challenge was not inducing overt pathology but was rather associated with a low histopathological score (S3 Fig).

Altogether, these data suggest that vaccination influenced the T-cell response in the lungs after influenza challenge. Mice immunized with SAM(NP) vaccines showed a rapid and enhanced recruitment of cytotoxic CD8^+^ T cells and polyfunctional CD4^+^ Th1 cells to the lungs that was associated with an efficient control of the virus, reduced lung lesions, and a significantly enhanced survival rate.

### Co-administration of the SAM(M1-NP) vaccine with monovalent inactivated influenza vaccine

An ideal cross-protective influenza vaccine should induce both humoral responses versus the surface HA antigen and T-cell responses against the internal conserved influenza antigens (NP and M1) [45, 46]. Therefore, we evaluated the possibility to use RNA-based SAM(M1-NP) vaccine in combination with a monovalent inactivated influenza vaccine (MIIV) derived from A/California/7/2009 (H1N1) virus (Cal/H1N1). BALB/c mice were immunized i.m. twice, eight weeks apart, with 0.1 μg of SAM(M1-NP) or SAM(GFP) control vector in LNP, combined with a suboptimal dose of MIIV (0.1 μg) chosen to assess the possible synergy with SAM vaccines. PBS-treated and mice pre-exposed to a low dose of PR8 influenza virus were used as negative and positive control, respectively. One month after the last immunization, mice were infected with 10-fold the lethal dose of PR8 virus to increase the stringency of the model and were monitored for 14 days after infection.

**Fig 7:**
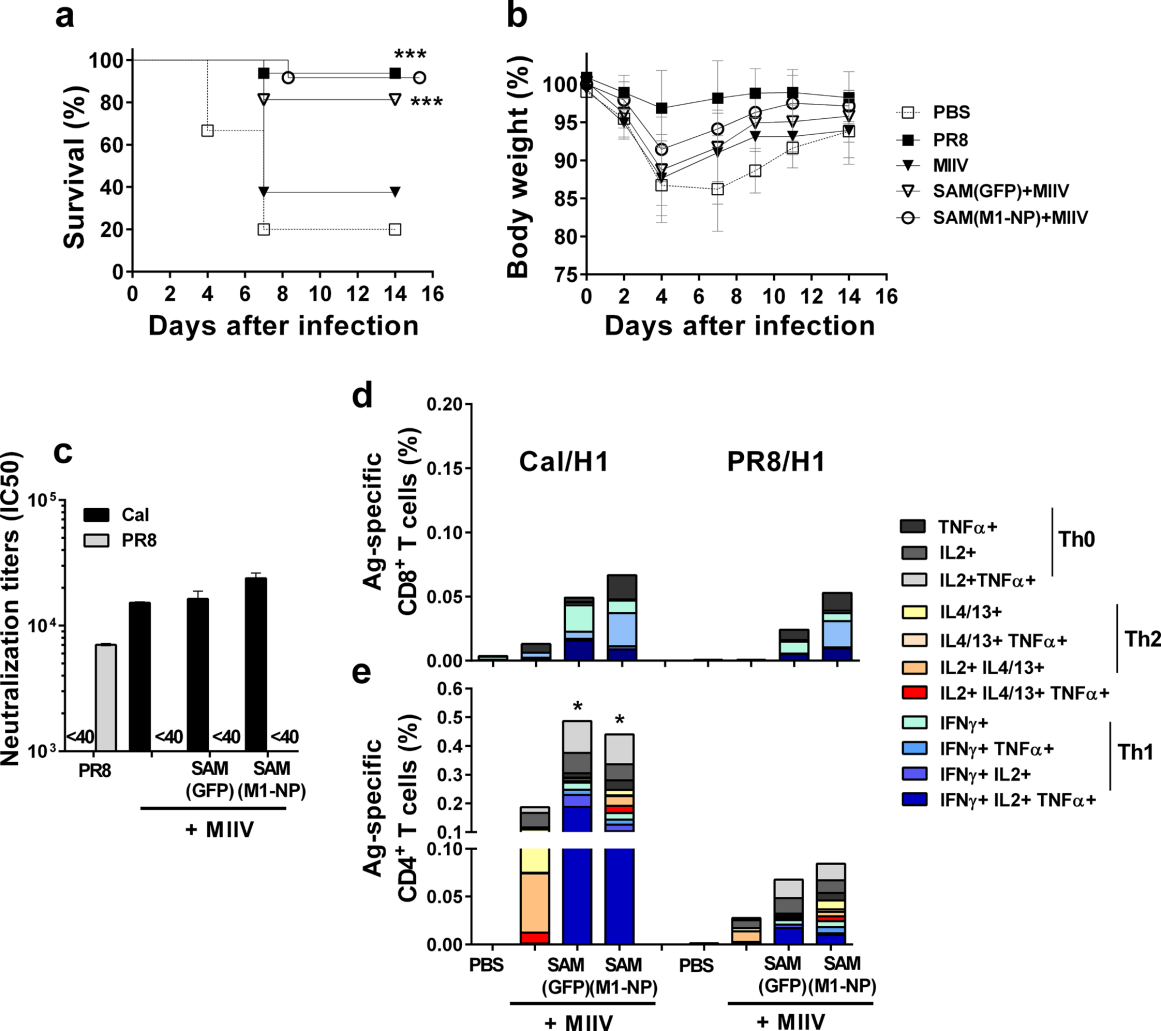
Addition of SAM vaccines enhanced the protection provided by MIIV alone in a heterologous influenza challenge model. BALB/c mice (n = 20) were immunized i.m. twice, 8 weeks apart, with 0.1 μg of SAM(M1-NP) or SAM(GFP) in combination with 0.1 μg of MIIV (Cal/H1N1). Four weeks after the last injection, mice were challenged with 10-fold the lethal dose of heterologous influenza PR8 virus. Mice were monitored for survival (a) and body weight loss (b) for 14 days after infection. Data show mean of single mice ± SD. Data are derived from two separate and merged experiments. Statistical analyses were performed using the Log rank analysis (Mantel Cox test). ***p<0.001 compared to PBS. (c) Neutralizing titers against Cal and PR8 viruses in sera collected two weeks after the second immunization. (d, e) Ten days after the second immunization, the frequency of antigen-specific cytokine-secreting CD8+ (d) or CD4+ (e) T cells was determined by flow cytometry on splenocytes stimulated *in vitro* with a Cal/H1 peptide pool (d, e) or a PR8/H1 peptide pool (d, e). Data are derived from two independent and merged experiments. Statistical analyses with the Mann-Whitney U test were performed on total cytokines. *p<0.05 compared to MIIV.

SAM(M1-NP)+MIIV-immunized mice showed a survival rate significantly increased compared to PBS treated mice with 91% and 20%, respectively, while MIIV alone provided only partial protection in these experimental conditions (37% of survival) (Fig 7A). All immunized animals showed signs of disease during the course of the observation, with a transient weight loss peaking four days after infection (Fig 7B). To our surprise, 80% of the animals in the SAM(GFP)+MIIV vector control group survived the infection, although we previously showed that SAM(GFP) alone did not induce H1-specific immune responses nor did it protect mice against a challenge with the PR8 virus [30].

To investigate a possible adjuvant effect [47, 48] of the ssRNA vector *in vivo*, we compared the adaptive immune responses induced by the different vaccine combinations by measuring antigen-specific functional antibody titers (Fig 7C) and T-cell frequencies (Fig 7D and 7E) two weeks after the second immunization. Virus neutralization titers against A/California/7/2009 (H1N1) vaccine strain ranged from 1.5x10^4^ to 2x10^4^, and were not significantly different in sera from mice immunized with SAM(M1-NP)+MIIV or SAM(GFP)+MIIV and MIIV alone, while no neutralizing activity was found against the PR8 virus (Fig 7C), confirming previous observations [30]. In contrast, combining SAM(M1-NP) or SAM(GFP) with MIIV resulted in increased frequencies of Cal/H1 vaccine-specific and PR8/H1 cross-reactive CD8^+^ and CD4^+^ T cells compared to MIIV (Fig 7D and 7E). H1-specific CD8^+^ T cells showed a polyfunctional effector phenotype consisting of combinations of IFN-γ and TNF-α. Moreover, the co-administration of SAM replicons with MIIV shifted the usual Th0/Th2 phenotype elicited by MIIV and characterized by the production of IL-13/IL-4, to a Th0/Th1 profile dominated by the production of IFN-γ/TNF-α/IL-2 and IL-2/TNF-α. The similar T helper pattern observed when combining SAM vectors encoding M1-NP or GFP antigens with MIIV suggests that the polarization of the T-cell response was due to the replicon *per se*, and was likely not antigen-dependent. Finally, NP- and M1-specific T-cell responses were normally observed in mice immunized with SAM(M1-NP)+MIIV (S4 Fig).

These results demonstrated that co-administration of SAM(M1-NP) and MIIV induced broader immunity resulting in an enhanced protection against heterologous influenza viruses compared to MIIV alone. This is also the first evidence that the combination of ssRNA vectors and protein-based vaccines might be feasible to improve the efficacy of current seasonal and pandemic influenza vaccines.

## Discussion

There is evidence in humans that T-cell responses, induced by natural exposure to influenza virus and directed toward the internal conserved antigens (NP and M1), can provide cross-protection and limit the severity of the disease [49, 50]. In this study, we assessed the immunogenicity and efficacy of self-amplifying mRNA vectors encoding NP and M1 antigens, separately or in combination, formulated with synthetic lipid nanoparticles. All SAM vaccines were immunogenic in BALB/c mice, inducing NP-specific cytotoxic CD8^+^ T cells and CD4^+^ Th1 cells and M1-specific CD4^+^ but not CD8^+^ T cells. SAM vaccines induced antigen-specific T_EM_ and T_CM_ cell subsets, both of which are important for long-lasting protection, confirming previous observations obtained with RNA vaccines [51]. Mice immunized with SAM vaccines expressing NP alone or in combination with M1 were protected from homologous and heterosubtypic influenza challenge. NP-specific CD8^+^ T cells infiltrating the lungs of these mice showed characteristics of cytotoxic and effector T cells, which correlated with a decrease in lung viral titers and lower immunopathology scores. Finally, we demonstrated that co-formulating ssRNA SAM vaccines with MIIV subunit protein brought the benefit of each platform (NP-/M1-specific T cells and HA-specific functional antibody), shifted the polarization of HA-specific CD4^+^ T cells from a Th0/Th2 to a Th0/Th1 phenotype, elicited HA-specific cytotoxic CD8^+^ T cells, and increased protection against the heterologous PR8 virus.

With the present study, we extended previous knowledge on the immunogenicity and protective efficacy of plasmid DNA or dsDNA viral vectors encoding NP and M1 antigens [8, 12, 17, 19, 52] to ssRNA SAM vectors. We showed that immune responses directed towards NP were essential to protect mice against homologous PR8 virus, confirming previous observations [12], since mice immunized with SAM(NP) alone or in combination with M1 showed comparable survival rates. In contrast, mice immunized with SAM(M1) showed T-cell responses limited to the CD4 subset, in agreement with recent observations obtained with DNA or recombinant vaccinia virus vaccines expressing M1 [42], and were not protected. Furthermore, all SAM vaccines provided complete protection against HK68 heterosubtypic virus, suggesting that cross-reactive T-cells were induced by the vaccines due to the high amino acid homology of the NP (94%) and M1 (98%) antigens between PR8 and HK68 viruses. While M1 seemed not essential in the mouse model of influenza, there are several lines of evidence in humans demonstrating that M1-specific T cells [49, 50, 53–56] have the ability to provide cross-protection [57, 58, 59]. CD8^+^ T cells play a crucial role in virus clearance, however, cytotoxic responses in both mice and humans are usually focused only on a fraction of immunogenic epitopes eliciting either immunodominant or subdominant responses [60]. In influenza virus-infected C57BL/6 mice, NP_366-374_- and PA_224-233_-specific CD8^+^ T-cell responses are dominant [61], while the immune-dominance of M1_58-66_–specific cytotoxic T cells has been well established in HLA-A2^+^ individuals [59], supporting the approach of including M1 in a universal influenza vaccine with broad cross-protective immunity. Clinical studies using the modified vaccinia virus Ankara (MVA) vector encoding NP and M1 (MVA-NP+M1) were conducted and provided the first evidence of safety and clinical efficacy of a T-cell-based influenza vaccine [62, 63]. Considering the correlation between preclinical and clinical data obtained with the MVA-NP+M1 vaccine, we are confident that SAM(M1-NP) represent an alternative approach for a universal influenza vaccine worth to be tested in humans.

An ideal universal influenza vaccine should induce neutralizing antibodies toward surface-exposed antigens such as HA and NA to reduce host cell infection, as well as T cells against the conserved internal antigens to eliminate influenza-infected cells [46] and provide broader protection in case of a HA missmatch between the vaccine and the circulating strains. We tested this approach by combining our lead candidate SAM(M1-NP) with MIIV and demonstrated that this vaccination regimen induced functional antibody responses to HA and T-cell responses to NP and M1 antigens. These observations are in line with reports on the co-administration of MVA-NP+M1 and a trivalent inactivated influenza vaccine in preclinical [64] and clinical studies [45]. We recently demonstrated that H1-specific CD4^+^ Th1 cells and cytotoxic CD8^+^ T cells also played a role in the protection against a heterologous influenza strain [30]. Interestingly, co-administration of ssRNA SAM, regardless of the antigen encoded by the vector (GFP, M1-NP), acted as an adjuvant for the subunit MIIV vaccine and elicited H1-specific effector CD8^+^ T cells, increased the magnitude of H1-specific CD4^+^ T cells, and shifted their polarization towards a Th1 phenotype. It is unclear if such an adjuvant effect is specific to ssRNA SAM vectors, or if it is shared with dsDNA vectors like MVA, as the combination of a seasonal vaccine with an MVA vector expressing an unrelated antigen was not reported [64]. In any case, the immune-modulatory effect of RNA through the engagement of Toll- and NOD-like receptors has been widely described in literature (see [65] for review). Considering the efficacy of SAM(HA) [30] and SAM(M1-NP) vaccines in mice, both vectors might be combined in a unique vaccine able to induce both T cell- and B cell-mediated immunity. Future studies are needed to address the immunogenicity and efficacy of such a combination.

In this study, we offer an alternative vaccine platform technology, based on ssRNA viral vectors, to deliver conserved influenza antigens and induce protective immune responses, similar to other nucleic acid-based vaccines such as mRNA [26], plasmid DNA [12, 15–17], or dsDNA viral vectors [14, 18, 19, 42]. However, the SAM technology combines the positive immunological attributes of DNA and viral vector-based vaccines, while potentially overcoming many of their limitations [66]. For example, the production of SAM vaccines does not require biological systems, is cell-free, rapid and highly scalable, facilitating a rapid response to emerging pathogens [29]. Only 8 days were needed to synthesize a prototype SAM(H7) vaccine against emerging H7N9 in response to the 2013 outbreak in China [29], while the cell-based production of a MVA vaccine took about 6–12 weeks [67]. Second, low doses of SAM vaccines elicit potent humoral and T-cell responses in small animals as well as in non-human primates without the risk of genome integration or anti-vector immunity [27, 28, 31, 68]. A vaccine platform based on the SAM^®^ technology that can induce a broad spectrum of immune responses, in addition to meeting production requirements, might be beneficial for the development of a broad-spectrum universal influenza vaccine.

## Supporting Information

S1 Fig SAM(NP) and SAM(M1) vaccines induce antigen-specific IgG. BALB/c mice (n = 24) were immunized i.m. twice, 8 weeks apart, with 0.1 μg of SAM(NP), SAM(M1), SAM(M1-NP), or with 0.2 μg of SAM(NP)+SAM(M1). Sera were collected 3 weeks after the first immunization (post 1) and 2 weeks after the second (post 2) to determine (a) NP- and M1-specific IgG titers by ELISA and (b) PR8 virus neutralization titers. Statistical analyses were performed using the Mann-Whitney U test. **p<0.01 compared to SAM(NP) or SAM(M1).
(TIF)

S2 Fig CD4 T-cell responses in lungs after influenza challenge. BALB/c mice were immunized i.m. twice, 8 weeks apart, with PBS, SAM(NP), SAM(M1), SAM(NP)+SAM(M1) or SAM(M1-NP), and four weeks after the second immunization, they were infected with PR8 virus. At day 0, 3, 6 and 17 after challenge, the cytokine profile of lung-derived CD4 T cells were assessed by flow cytometry after *in vitro* stimulation with medium (m), recombinant NP protein (NP), or M1 peptide pool (M1). Data are derived from two independent and merged experiments. Statistical analyses were performed using the Mann-Whitney U test comparing each immunization group with the PBS group for each stimulus (*) and comparing the different stimuli within the immunization group (•). **p<0.01; •*p* <0.05.
(TIF)

S3 Fig Characterization of lung pathology following influenza challenge. BALB/c mice (n = 24) were immunized i.m. twice 8 weeks apart with PBS, 0.1 μg of SAM(NP), SAM(M1), SAM(M1-NP), or with 0.2 μg of SAM(NP)+SAM(M1). Four weeks after the second immunization, mice were infected with a lethal dose of PR8 virus. Lungs were collected after infection to evaluate tissue damage (S1 Materials and Methods). (a) Clinical scores were given for severity as follows: 0, normal lung; 1 mild and/or scattered foci of inflammation; 2 moderate and/or several foci of inflammation; 3 severe and/or diffuse inflammation. Three mice per group per time point were scored; data show mean ± SD. (b) Representative images of H&E stained sections. Black arrows indicate areas of cellular infiltration and red arrow highlights foci of edema. Magnifications of the alveoli show epithelial denudation and cell infiltration, vessel magnifications demonstrate a severe and mild perivascular cuffing in PBS and SAM(M1-NP) lungs, respectively.
(TIF)

S4 Fig MIIV+SAM(M1-NP) co-administration induces T-cell responses versus NP and M1 antigens. BALB/c mice (n = 20) were immunized i.m. twice, 8 weeks apart, with 0.1 μg of SAM(M1-NP) or SAM(GFP) in combination with 0.1 μg of MIIV. Ten days after the second immunization, the frequency of antigen-specific cytokine-secreting CD8^+^ (a) or CD4^+^ (b, c) T cells was determined by flow cytometry on splenocytes stimulated *in vitro* with NP_147-155_ peptide (a), recombinant NP protein (b), or M1 peptide pool (c). Data are derived from two independent and merged experiments. Statistical analyses were performed using the Mann-Whitney U test. *p<0.05 compared to MIIV.
(TIF)

S1 Materials and Methods Supplementary Materials and Methods. (DOCX)
