# Supplementary figures and images for: Self-Amplifying mRNA Vaccines Expressing Multiple Conserved Influenza Antigens Confer Protection against Homologous and Heterosubtypic Viral Challenge
Source: PLoS One. 2016 Aug 15;11(8):e0161193. doi: 10.1371/journal.pone.0161193 (PMC4985159; doi:10.1371/journal.pone.0161193)

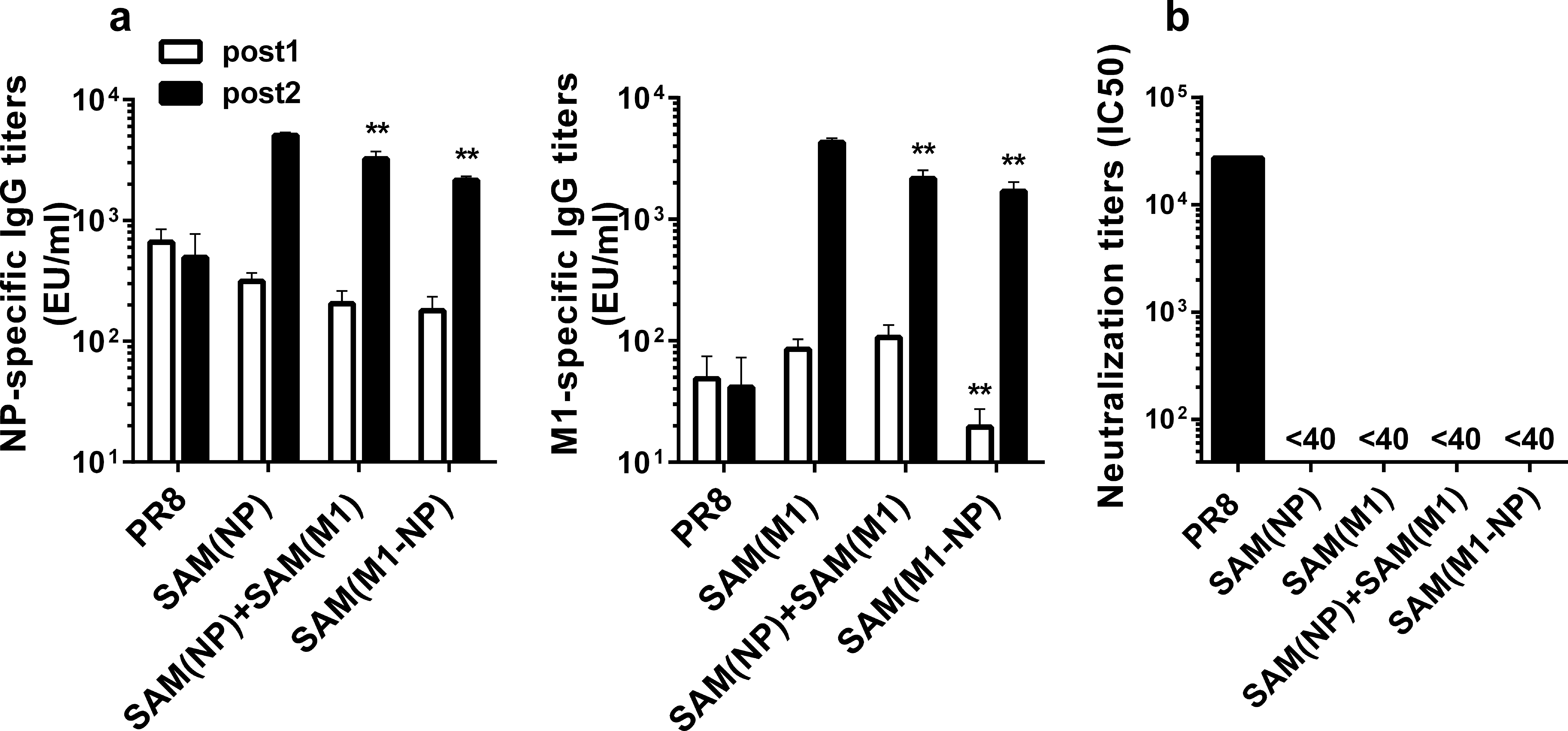

Supplement: S1 Fig — BALB/c mice (n = 24) were immunized i.m. twice, 8 weeks apart, with 0.1 μg of SAM(NP), SAM(M1), SAM(M1-NP), or with 0.2 μg of SAM(NP)+SAM(M1). Sera were collected 3 weeks after the first immunization (post 1) and 2 weeks after the second (post 2) to determine (a) NP- and M1-specific IgG titers by ELISA and (b) PR8 virus neutralization titers. Statistical analyses were performed using the Mann-Whitney U test. **p<0.01 compared to SAM(NP) or SAM(M1). (TIF) [file pone.0161193.s001.tif]

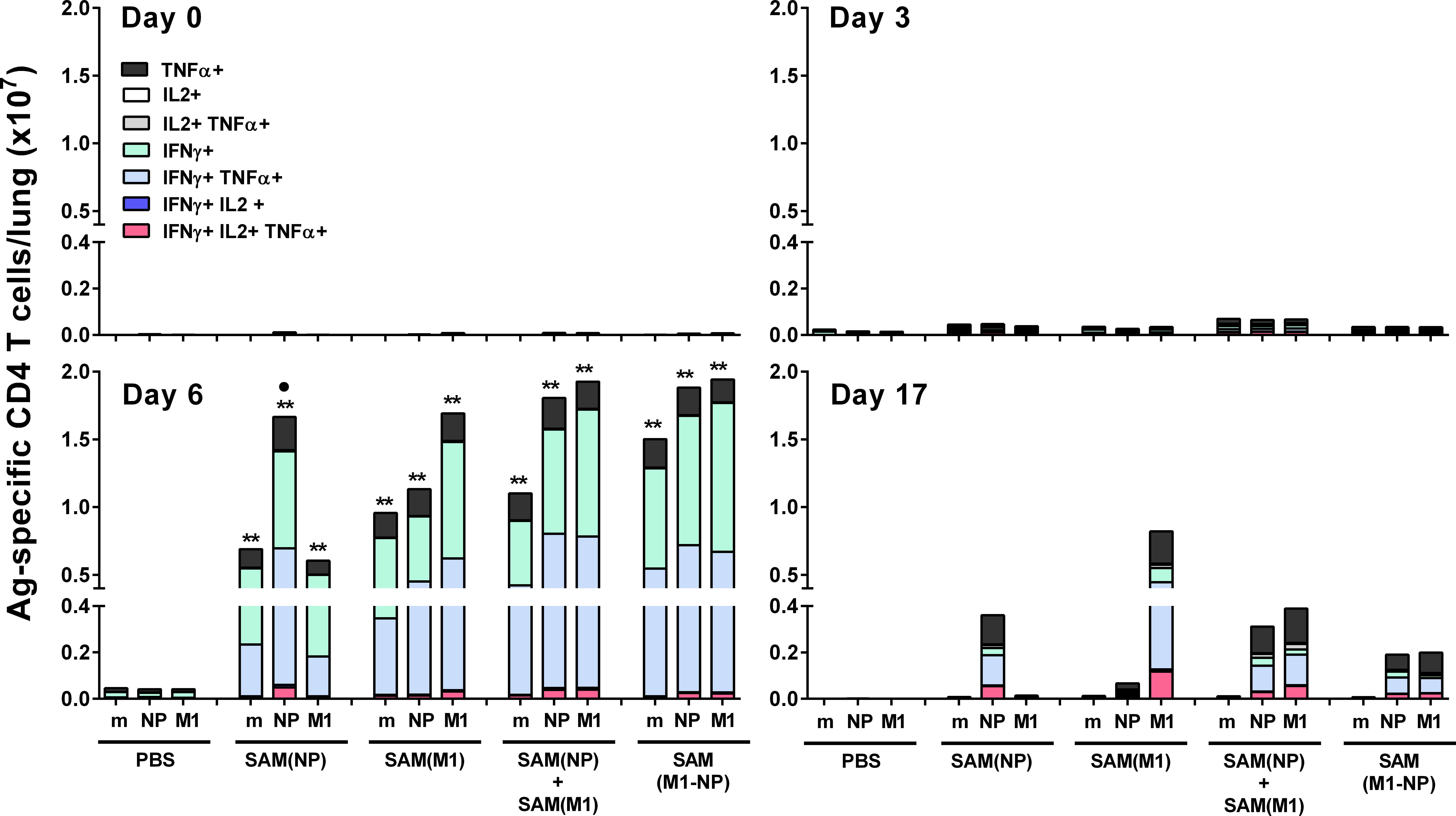

Supplement: S2 Fig — BALB/c mice were immunized i.m. twice, 8 weeks apart, with PBS, SAM(NP), SAM(M1), SAM(NP)+SAM(M1) or SAM(M1-NP), and four weeks after the second immunization, they were infected with PR8 virus. At day 0, 3, 6 and 17 after challenge, the cytokine profile of lung-derived CD4 T cells were assessed by flow cytometry after in vitro stimulation with medium (m), recombinant NP protein (NP), or M1 peptide pool (M1). Data are derived from two independent and merged experiments. Statistical analyses were performed using the Mann-Whitney U test comparing each immunization group with the PBS group for each stimulus (*) and comparing the different stimuli within the immunization group (•). **p<0.01; •p <0.05. (TIF) [file pone.0161193.s002.tif]

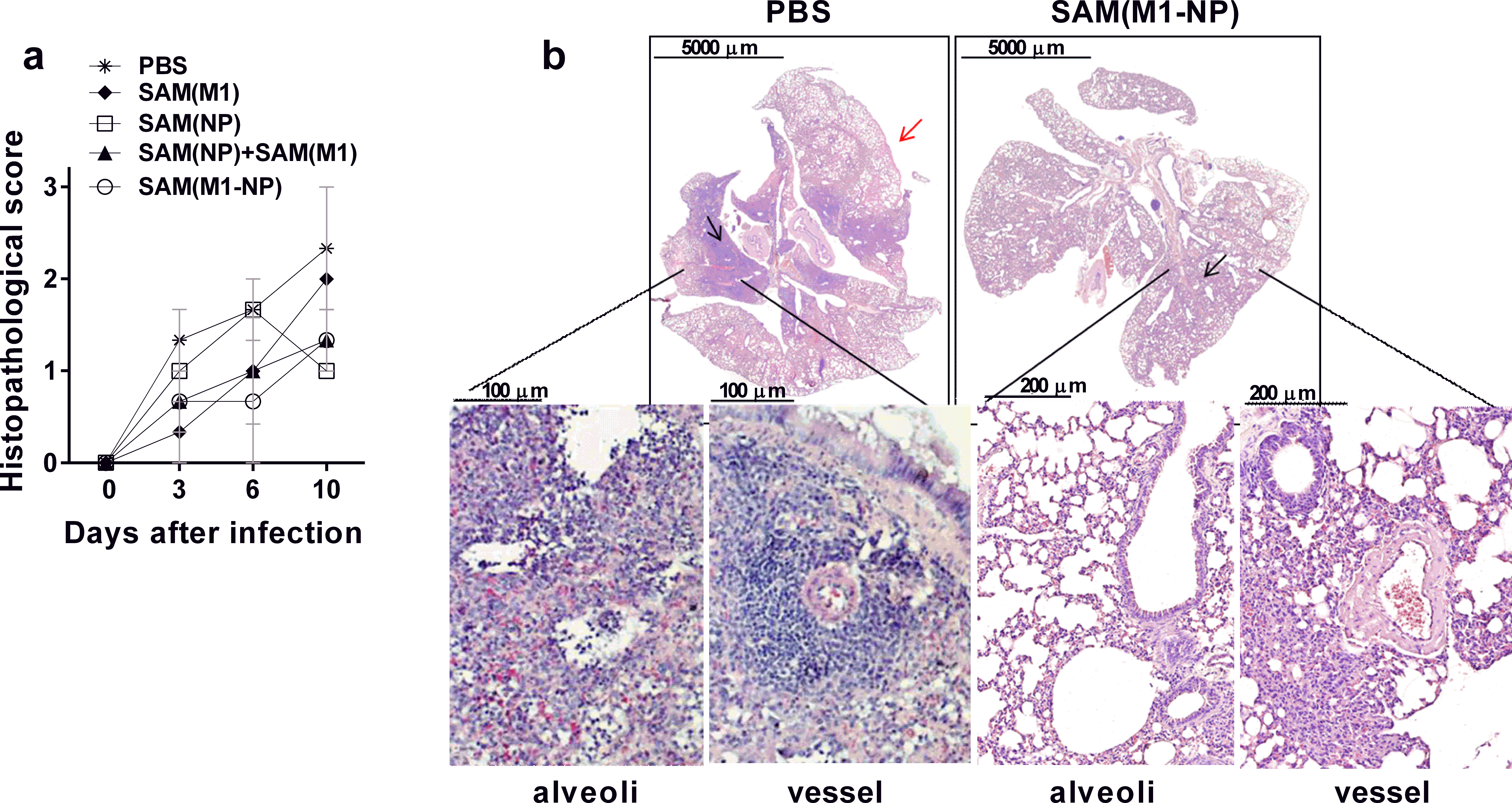

Supplement: S3 Fig — BALB/c mice (n = 24) were immunized i.m. twice 8 weeks apart with PBS, 0.1 μg of SAM(NP), SAM(M1), SAM(M1-NP), or with 0.2 μg of SAM(NP)+SAM(M1). Four weeks after the second immunization, mice were infected with a lethal dose of PR8 virus. Lungs were collected after infection to evaluate tissue damage (S1 Materials and Methods). (a) Clinical scores were given for severity as follows: 0, normal lung; 1 mild and/or scattered foci of inflammation; 2 moderate and/or several foci of inflammation; 3 severe and/or diffuse inflammation. Three mice per group per time point were scored; data show mean ± SD. (b) Representative images of H&E stained sections. Black arrows indicate areas of cellular infiltration and red arrow highlights foci of edema. Magnifications of the alveoli show epithelial denudation and cell infiltration, vessel magnifications demonstrate a severe and mild perivascular cuffing in PBS and SAM(M1-NP) lungs, respectively. (TIF) [file pone.0161193.s003.tif]

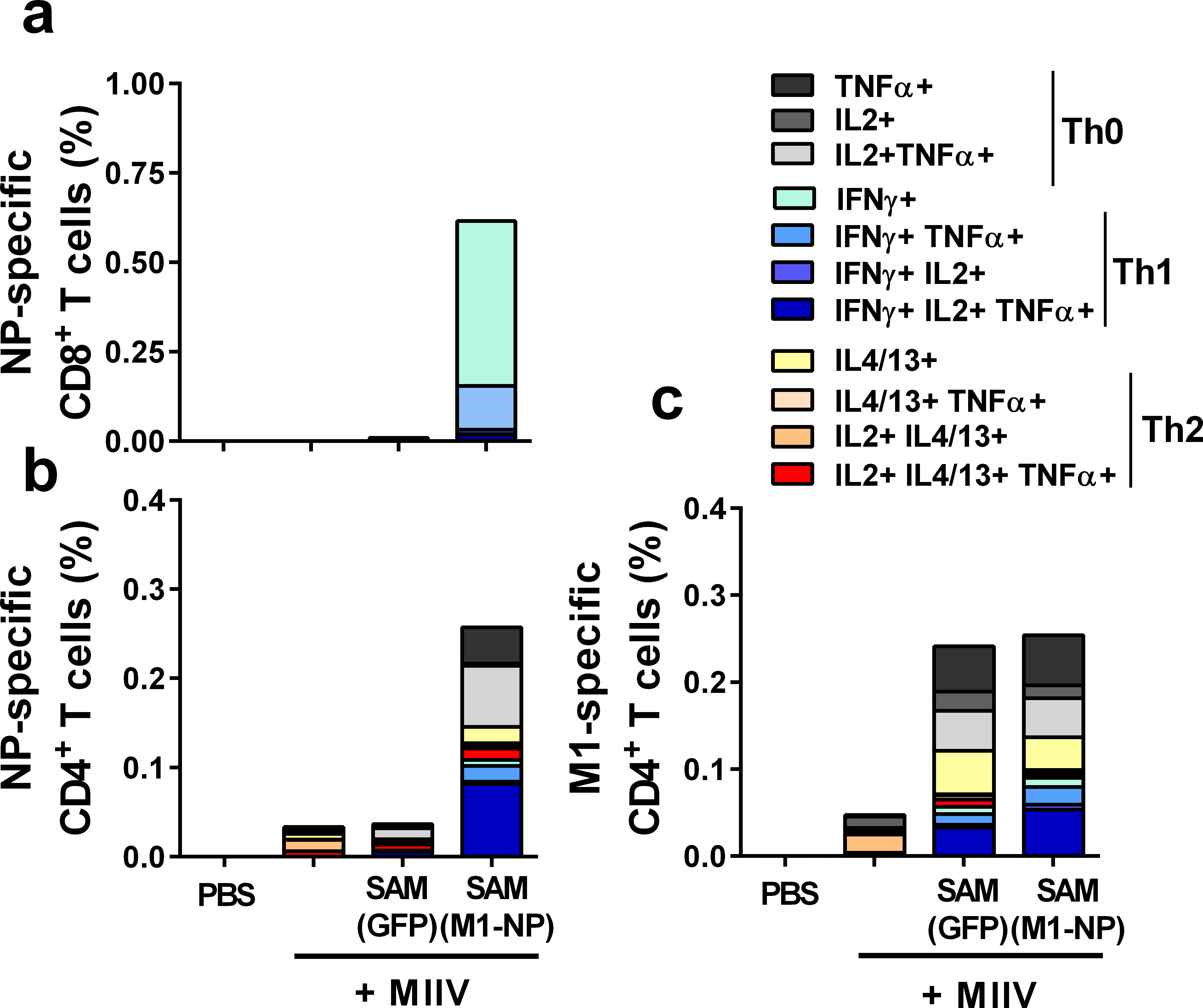

Supplement: S4 Fig — BALB/c mice (n = 20) were immunized i.m. twice, 8 weeks apart, with 0.1 μg of SAM(M1-NP) or SAM(GFP) in combination with 0.1 μg of MIIV. Ten days after the second immunization, the frequency of antigen-specific cytokine-secreting CD8+ (a) or CD4+ (b, c) T cells was determined by flow cytometry on splenocytes stimulated in vitro with NP147-155 peptide (a), recombinant NP protein (b), or M1 peptide pool (c). Data are derived from two independent and merged experiments. Statistical analyses were performed using the Mann-Whitney U test. *p<0.05 compared to MIIV. (TIF) [file pone.0161193.s004.tif]
