# Supplementary material for: Self-Amplifying mRNA Vaccines Expressing Multiple Conserved Influenza Antigens Confer Protection against Homologous and Heterosubtypic Viral Challenge
Source: PLoS One. 2016 Aug 15;11(8):e0161193. doi: 10.1371/journal.pone.0161193 (PMC4985159; doi:10.1371/journal.pone.0161193)
Supplement: S1 Materials and Methods — (DOCX) [file pone.0161193.s005.docx]

**Supplementary Material and Methods**

**Histopathological examination**

Lung samples were fixed in 4% formaldehyde, paraffin-embedded, then cut 4 μm thick and stained with hematoxylin and eosin (H&E). Routine histological examination was performed blind by a veterinary pathologist. Histological changes were evaluated and lesions were scored for severity as follows: 0, normal lung; 1, mild and/or scattered foci of inflammation; 2, moderate and/or several foci of inflammation; 3, severe and/or diffuse inflammation (e.g. lobar).

Tissues were also analyzed according to the distribution and characteristics of lesions: presence of intra-bronchial/intra-bronchiolar exudation, epithelial denudation, intraepithelial abscesses, degeneration and thickening of alveolar walls, intra-alveolar edema and infiltration, hyaline membranes, proliferation of pneumocytes, perivascular and peribronchial cuffing, arteriolar degeneration.
